# Supplementary material for: Integrated Proteomics and Metabolomics Link Acne to the Action Mechanisms of Cryptotanshinone Intervention
Source: Front Pharmacol. 2021 Sep 1;12:700696. doi: 10.3389/fphar.2021.700696 (PMC8440807; doi:10.3389/fphar.2021.700696)
Supplement: Supplementary file 1 [file DataSheet1.ZIP › Supplementary table 1-10 and material 1-3/Material 3-Metlib-MSMS/POS-Metlib-MSMS/Metlib-MSMS/M532T179_forward/0.957,1-O-Octadecyl-sn-glyceryl-3-phosphorylcholine,(M+Na)+.pdf]

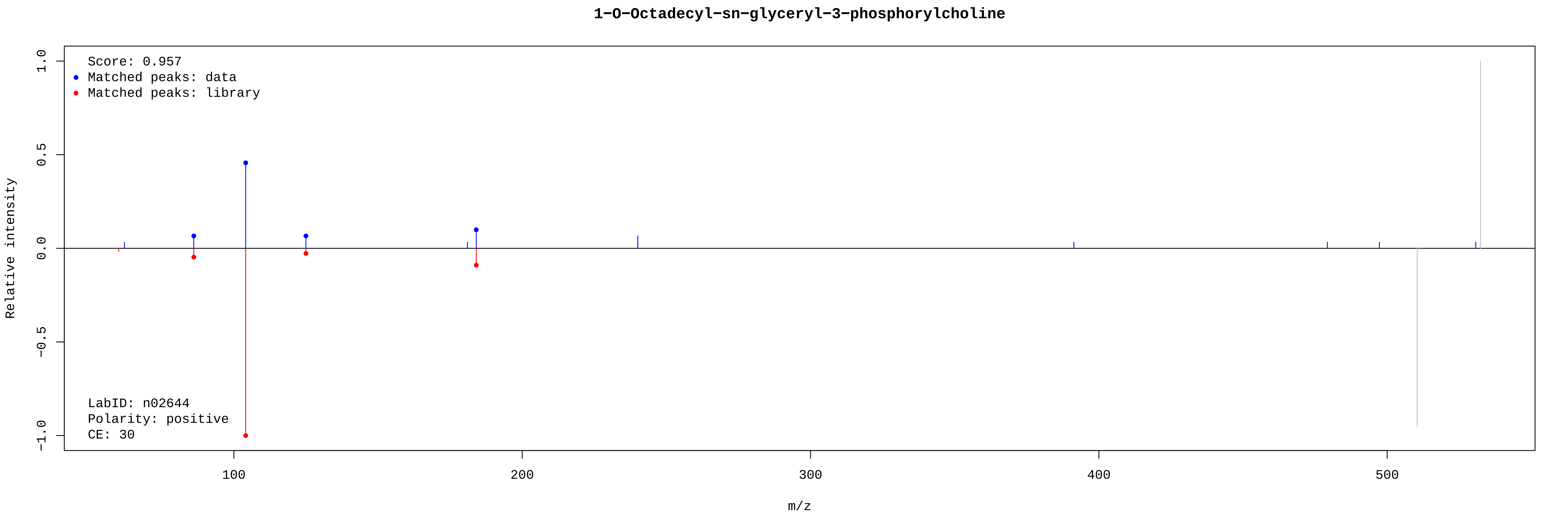

1-O-Octadecyl-sn-glyceryl-3-phosphorylcholine
Score: 0.957
Matched peaks: data
Matched peaks: library
LabID: n02644
Polarity: positive
CE: 30
Relative intensity
m/z
1.0
0.5
0.0
-0.5
-1.0
100
200
300
400
500
